# The Transition between Telomerase and ALT Mechanisms in Hodgkin Lymphoma and Its Predictive Value in Clinical Outcomes

**DOI:** 10.3390/cancers10060169

**Published:** 2018-05-30

**Authors:** Radhia M’kacher, Corina Cuceu, Mustafa Al Jawhari, Luc Morat, Monika Frenzel, Grace Shim, Aude Lenain, William M. Hempel, Steffen Junker, Theodore Girinsky, Bruno Colicchio, Alain Dieterlen, Leonhard Heidingsfelder, Claire Borie, Noufissa Oudrhiri, Annelise Bennaceur-Griscelli, Olivier Moralès, Sarah Renaud, Zoé Van de Wyngaert, Eric Jeandidier, Nadira Delhem, Patrice Carde

**Affiliations:** 1 Laboratoire de Radiobiologie et d’Oncologie, IRCM/DSV/CEA, 92265 Fontenay aux Roses, France; cuceu_corina@yahoo.com (C.C.); mustafa.aljawhari@hotmail.fr (M.A.J.); luc.morat@cea.fr (L.M.); monika.frenzel@hotmail.com (M.F.); graceshim1@gmail.com (G.S.); audelenain@yahoo.fr (A.L.); williamhempel824@gmail.com (W.M.H.); 2 Cell Environment, DNA Damages R&D, Oncology Section, 75020 Paris, France; 3 Institute of Biomedicine, University of Aarhus, DK-8000 Aarhus C, Denmark; sjunker@biomed.au.dk; 4 Department of Radiation Therapy, Gustave Roussy Cancer Campus, 94808 Villejuif, France; theogirinsky@me.com; 5 IRIMAS, Institut de Recherche en Informatique, Mathématiques, Automatique et Signal, Université de Haute-Alsace, 68093 Mulhouse, France; bruno.colicchio@uha.fr (B.C.); alain.dieterlen@uha.fr (A.D.); 6 MetaSystems GmbH, Robert-Bosch-Str. 6, D-68804 Altlussheim, Germany; lheidingsfelder@metasystems.de; 7 Université Paris Sud, Service d’hématologie moléculaire et cytogénétique Paul brousse CHU paris Sud, Inserm UMRS935, 94800 Villejuif, France; claire.borie@aphp.fr (C.B.); noufissa.oudrhiri@aphp.fr (N.O.); annelise.bennaceur@aphp.fr (A.B.-G.); 8 CNRS, Institut Pasteur de Lille, UMR 8161—Immunoregulation of Virus-induced Cancers Team, F-59000 Lille, France; olivier.morales@ibl.cnrs.fr (O.M.); sarah.renaud@ibl.cnrs.fr (S.R.); nadira.delhem@ibl.cnrs.fr (N.D.); 9 CHRU Lille Service des Maladies du Sang, Hopital Huriez, 59000 Lille, France; zoe.vdw@gmail.com; 10 Service de génétique, Groupe hospitalier de la région de Mulhouse Sud-Alsace, 68093 Mulhouse, France; jeandidiere@ghrmsa.fr; 11 Department of Medicine, Gustave Roussy Cancer Campus, 94808 Villejuif, France; dr.pcarde@gmail.com

**Keywords:** Hodgkin Lymphoma, telomerase, alternative lengthening of telomeres, EBV

## Abstract

*Background*: We analyzed telomere maintenance mechanisms (TMMs) in lymph node samples from HL patients treated with standard therapy. The TMMs correlated with clinical outcomes of patients. *Materials and Methods*: Lymph node biopsies obtained from 38 HL patients and 24 patients with lymphadenitis were included in this study. Seven HL cell lines were used as in vitro models. Telomerase activity (TA) was assessed by TRAP assay and verified through hTERT immunofluorescence expression; alternative telomere lengthening (ALT) was also assessed, along with EBV status. *Results*: Both TA and ALT mechanisms were present in HL lymph nodes. Our findings were reproduced in HL cell lines. The highest levels of TA were expressed in CD30−/CD15− cells. Small cells were identified with ALT and TA. Hodgkin and Reed Sternberg cells contained high levels of PML bodies, but had very low hTERT expression. There was a significant correlation between overall survival (*p* < 10^−3^), event-free survival (*p* < 10^−4^), and freedom from progression (*p* < 10^−3^) and the presence of an ALT profile in lymph nodes of EBV+ patients. *Conclusion*: The presence of both types of TMMs in HL lymph nodes and in HL cell lines has not previously been reported. TMMs correlate with the treatment outcome of EBV+ HL patients.

## 1. Introduction

Hodgkin Lymphoma (HL) is a malignancy characterized by the presence of scarce malignant cells comprising only 1–2% of the total tumor burden. The malignant cells include multi-nucleated Reed–Sternberg (RS) cells and mono-nucleated Hodgkin cells, derived from germinal center B cells [1]. The cells carry rearranged and somatically mutated immunoglobulin genes [1,2] and are characterized by an extinguished B-cell phenotype [3,4].

Telomere shortening in malignant cells and peripheral blood lymphocytes of HL patients has been documented [5,6,7]. This dysfunction requires the activation of a telomere maintenance mechanism (TMM) to support immortalization. In most malignant cells, expression of the gene encoding telomerase is activated [8]. However, in some cases, telomeres are also elongated using telomerase-independent mechanisms, termed alternative lengthening of telomeres (ALT) [9]. Previous studies have demonstrated the presence of telomerase activity (TA) and/or ALT mechanisms in various tumor types. In contrast to cells with active telomerase, cells that use ALT are characterized by (a) intracellular heterogeneity in telomere length, ranging from very short to very long [8,10]; (b) ALT-associated promyelocytic leukemia (PML) bodies (APBs); (c) the presence of extrachromosomal telomeric repeats [9,11]; and (d) high levels of telomere sister chromatid exchanges (T-SCEs) [12]. Most tumors exhibit characteristics of just one of the TMMs. Nevertheless, reports have not generally clarified whether specific tumors, displaying ambiguous characteristics concerning telomere length, TA, and the presence of APBs used TA or ALT as a TMM [13,14,15,16]. Coexistence of ALT and telomerase has been demonstrated both in vitro [17,18,19] and in vivo [20,21,22]. Nevertheless, the ability of some cancer cells to “switch” from one to the other mechanism is still unclear [23]. Moreover, some tumors may lack telomerase and ALT [24].

The TMMs in HL tumor cells and established HL cell lines have only been investigated in a few papers, but the conclusions are discordant [25,26,27]. Norrback et al. [25] reported TA at low levels in 31 of 77 HL lymph nodes and at high levels in HL cell lines. Brousset et al. reported the presence of TA in only 2 of 20 HL lymph nodes [27].

Hence, in the absence of detectable TA, alternative telomerase-independent mechanism(s) for telomere maintenance in HL has been proposed.

In this study, we have reevaluated the TMMs in 38 lymph nodes of a homogeneous cohort of HL patients (82% stage I and 95% sclero-nodular) and seven established HL cell lines. We demonstrate, for the first time, the coexistence of both telomerase and ALT positive tumor cells in the same HL samples, suggesting that single cells do not exclusively use only one TMM. Moreover, TMM heterogeneity correlated with tumor-cell progression in HL. A poor clinical outcome was observed in patients with the dominant ALT mechanisms, particularly EBV+ patients.

## 2. Results

### 2.1. Telomerase Activity or ALT Pathway for Telomere Maintenance in HL Cell Lines

**Figure 1:**
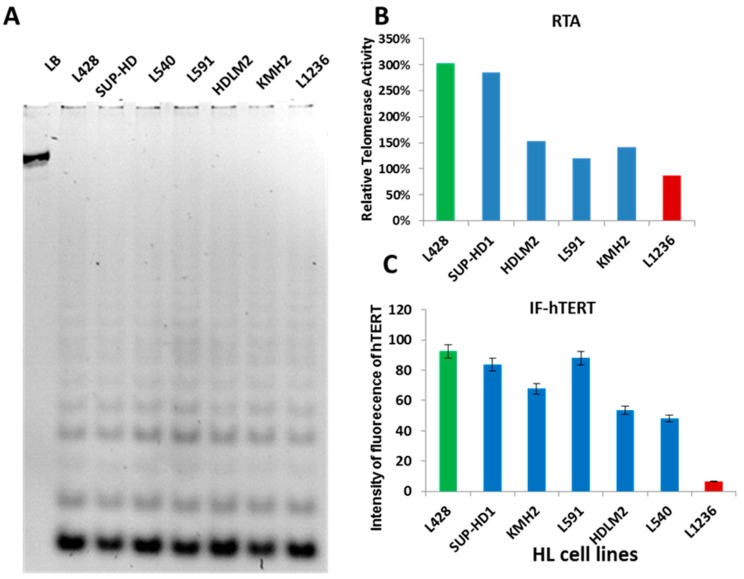
Telomerase expression in Hodgkin lynphoma (HL) cell lines. (**A**) PCR-based telomere repeat amplification protocol (TRAP) assay to determine the presence of telomerase activity (TA) in HL cell-lines. A lysis buffer (LB) serves as an internal control for the amplification, excluding false negatives. All HL cell lines expressed TA. (**B**) Histogram displaying the fold change of relative telomerase activity (RTA) in HL cell lines compared to CT high (positive control equal to 100%). (**C**) Quantification of the intensity of fluorecence of hTERT protein by imunofluorescence; 10,000 cells were scored. All data are representative of three independent experiments and expressed as the mean±standard error of the mean. The experiments were performed in triplicate.

First, we examined TA in seven HL cell lines by TRAP assay. TA was detected in all HL cell lines (Figure 1A) but relative TA levels varied between the cell lines (Figure 1B). In addition, a high copy number of *hTERT (5p15)* was observed in 3/7 HL cell lines with high TA levels, which was associated with hTERT breakpoint rearrangements without amplification (Figure S1). In the L1236 cell line, a deletion of one of the *hTERT* alleles was associated with a t(5;22) event (Figure S1). hTERT protein, detected by immunofluorescence, was expressed in all cell lines, although at different levels (Figure 1C), and, thus, corroborate our findings by the TRAP assay: L428, SUP-HD1, and L591 cells expressed high levels of hTERT protein, whereas L1236 cells expressed only low levels of hTERT, possibly due to deletion of an *hTERT* allele. These results demonstrate that telomerase is active in all HL cell lines, but relative levels vary between the cell lines.

**Figure 2:**
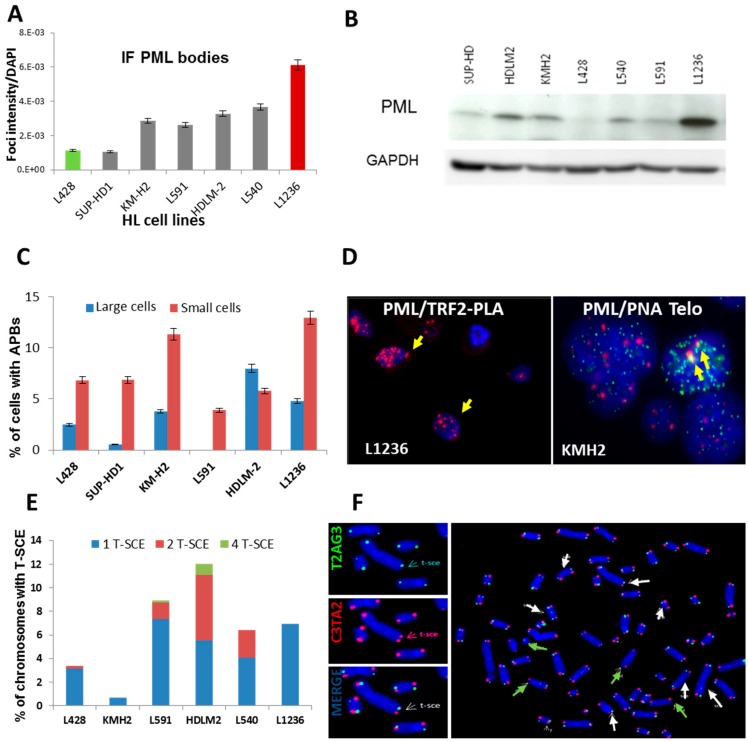
Charaterization of the alternative telomere lengthening (ALT) phenotype in HL cell lines. (**A**) Quantification of PML bodies in HL cell lines by immunofluorescence. Ten thousand cells were analyzed for each cell line. (**B**) Western blots of PML protein in HL cell lines. Glyceraldehyde-3-phosphate dehydrogenase (GAPDH) was used as a loading control. (**C**) Frequency of small and large cells with colocalization of TRF2 and PML by the PLA assay. (**D**) Representative cells with colocalization of PML and TRF2 by the PLA assay (yellow arrow) and the manual colocalization of PML (red) and PNA-telomeres (green) (yellow arrow) (40× magnification). (**E**) Quantification of T-SCE in chromosomes of HL cell lines after CO-FISH staining. Chromosomes with (i) one T-SCE event, (ii) with two T-SCE events assessed by simultaneously using both leading- and lagging-strand probes, and (iii) with four T-SCE events on both strands and on both the p and q arms were assessed. (**F**) Image of metaphases with T-SCE (white arrow) in KMH2 cells and telomere deletions (green arrow) (63× magnification).

The considerable heterogeneity of hTERT expression between the various HL cell lines and the presence of long heterogeneous telomeres, previously identified by Q-FISH [28], suggest that ALT mechanisms are also active in HL cell lines. Therefore, we analyzed ALT characteristics using co-localization of PML protein with telomeres/telomeric proteins to identify APBs [29] and telomeric sister exchanges (T-SCEs). First, PML bodies were quantified in HL cell lines by immunofluorescence (Figure 2A) and western blotting (Figure 2B). We further corroborated these data by FISH painting, which revealed a high copy number of *PML* in the L1236 cell line (Figure S2). Second, we used the proximity ligation assay (PLA) to detect APBs, the co-localization of telomeres and PML protein, via TRF2 signals. The distribution of APB foci in HL cell lines shown in Figure 2C demonstrates a high number of co-localization foci in small cells (Figure 2D). These data have been validated with manual identification of PML/PNA-telomeres (IF-FISH) (Figure S2B). Third, we used the CO-FISH technique to quantify T-SCEs, which are rare or absent in non-ALT cells [12]. HDLM2, L591, L540, and L1236 cell lines displayed a higher frequency of T-SCEs than did L428 and KMH2 cell lines (Figure 2E,F).

**Figure 3:**
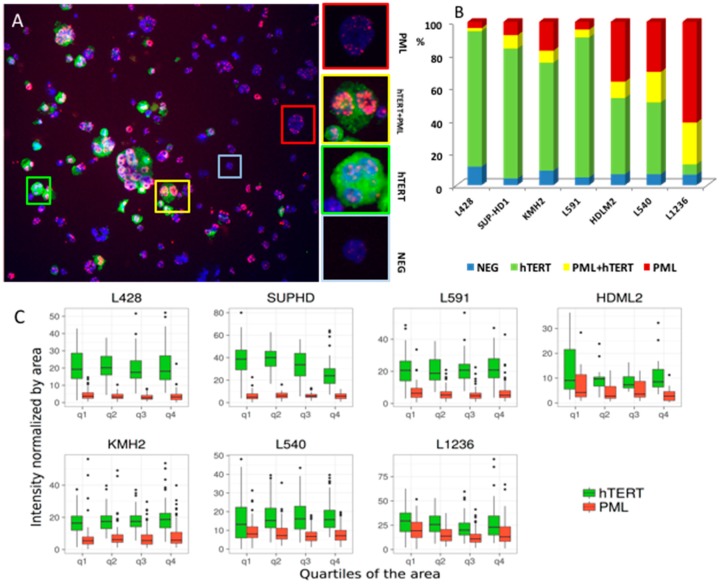
Telomerase and PML body expression in HL cell lines. (**A**) hTERT (green signal) and PML (red signal) expression divided HDLM2 cells into four classes: (i) Cells without any signal (blue frame), (ii) hTERT positive cells (green frame), (iii) cells with hTERT and PML expression (yellow frame), and (iv) cells with PML bodies (red frame) (10× magnification). (**B**) Scoring of the frequency of each type of cells according to this classification. Two thousand cells were scored for each cell line. (**C**) Fluorescence intensity was measured in the different cell types by automated quantification of the hTERT and PML signals. The intensity of fluorescence of hTERT and PML was normalized by the area of cells and results are represented according to the quartiles of the area of the cells. The median, 95% confidence interval, min and max are presented.

Overall, these data demonstrate coexistence of TA and ALT in HL cell lines. Immunofluorescence of PML bodies and hTERT protein revealed the presence of (1) cells with only hTERT expression, (2) cells with only PML expression, (3) cells exhibiting both hTERT and PML expression, (4) and cells without any expression (Figure 3A). The positive control for hTERT and PML immunofluorescence is depicted in Figure S3. The scoring of cells according to this classification revealed the presence of all four categories in all HL cell lines at different levels (Figure 3B). Interestingly, we demonstrated the coexistence of both telomerase and PML in the same cell line and in the same cells. The L428, SUP–HD1, and L591 cell lines (high TA) showed a high frequency of cells with hTERT expression. However, a large proportion of L1236 cells (low TA) showed a high frequency of cells with only PML expression (Figure 3B).

We next focused on the nature of cells exhibiting PML and/or hTERT proteins by investigating whether the PML protein may contribute to telomere surveillance during the transition between the small and/or Hodgkin cells to Reed-Sternberg cells. Given the heterogeneity in the size of the cells in the HL cell lines, we used automated fluorescence image analysis, allowing us to not only screen a large number of cells, but also to determine their size, based on their area. The distribution of fluorescence intensity of PML and hTERT were significantly different (*p* < 10^−2^ to *p* < 10^−16^) in all cells. hTERT and PML expression significantly correlated with the area of the cells. Small cells (1st quartile) exhibited a higher intensity of fluorescence for hTERT than large RS-like cells (4th quartile) (*p* = 0.02 to *p* = 001) except in L428 and SUP-HD cell lines. The intensity of PML inversely correlated with the intensity of hTERT, such that the intensity of PML in small cells was significantly lower than that observed in large cells in the L1236, HDLM2 and L540 cell lines (*p* = 0.02, *p* = 0.01 and *p* = 0.05 respectively) (Figure 3C).

### 2.2. Transition between TMMs According to Cell Phenotype

**Figure 4:**
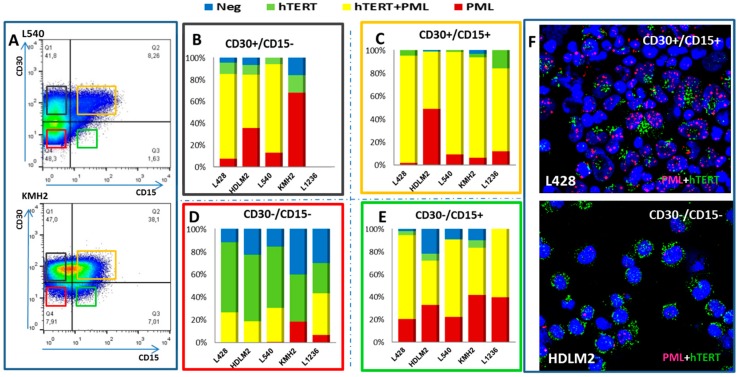
hTERT and PML expression in five HL cell lines according to CD15/CD30 phenotype. (**A**) Four subpopulations from each cell line were sorted according to CD30 and CD15 expression: CD30−/CD15−, CD30+/CD15−, CD30+/CD15+, CD30−/CD15+; (**B**) hTERT and PML expression in CD30+/CD15−; (**C**) in CD30+/CD15+; (**D**) in CD30−/CD15−; and (**E**) in CD30−/CD15+. Cells without any staining (blue), cells with only hTERT expression (green), cells with PML and hTERT expression (yellow), and cells with only PML expression (red). (**F**) Representative image of CD30+/CD15+ L428 cells and of CD30−/CD15− HDLM2 cells showing different types PML and hTER expression (40× magnification).

We determined the immunophenotypes of cells expressing hTERT and those with an ALT profile by performing fluorescence activation cell sorting after CD30 and CD15 staining of five HL cell lines (Figure 4A). Four sub-populations were isolated: CD30+/CD15− (Figure 4B), CD30+/CD15+ (Figure 4C), CD30−/CD15− (Figure 4D), and CD30−/CD15+ (Figure 4E). We observed the highest frequency of telomerase expressing cells in the CD30−/CD15− population, irrespective of the origin of cell line (Figure 4D). Nevertheless, high levels of CD30+/CD15+ cells exhibited both hTERT and PML expression (Figure 4C). Interestingly, we observed a high frequency of cells without staining in CD30−/CD15− cells (Figure 4D,F). Decreasing hTERT expression was associated with increasing PML expression during the transition of HL cells.

### 2.3. Primary Malignant HRS Cells Activate a Switch between Telomerase and ALT Mechanisms

Our findings on TMMs in HL cell lines suggest that tumors in HL patients may contain cells without any TMM expression, some cells expressing high telomerase, others expressing characteristics of ALT, and some expressing both types of telomere-elongating characteristics. We examined TMMs in 38 HL lymph nodes and 24 lymph nodes of lymphadenitis patients.

**Figure 5:**
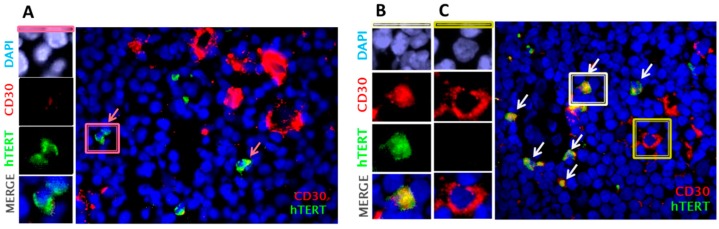
Immunofluorescence analysis of CD30 (red) and hTERT protein (green) of cells in lymph nodes of two different HL patients demonstrate (**A**) the presence of very high levels of hTERT in small cells lacking CD30 (pink arrows), (40× magnification), (**B**) co-localization of hTERT and CD30 expression in Hodgkin cells, (white arrows), and (**C**) the absence of hTERT expression in HRS cells (yellow frame) (40× magnification).

First, we performed immunofluorescence staining of CD30, one of the clinical hallmarks of HL tumor cells, and hTERT protein to determine the phenotype of cells expressing hTERT in HL lymph nodes. Figure 5A shows the presence of small CD30− cells with high levels of telomerase. Nevertheless, we observed co-localization of hTERT and CD30 in small cells (Figure 5B). HRS cells were characterized by low or no telomerase expression (Figure 5C). Suppression or low levels of hTERT was accompanied by the de novo expression of CD30, suggesting that Hodgkin cells express higher levels of telomerase than Reed-Sternberg cells, which display only very weak hTERT signals.

**Figure 6:**
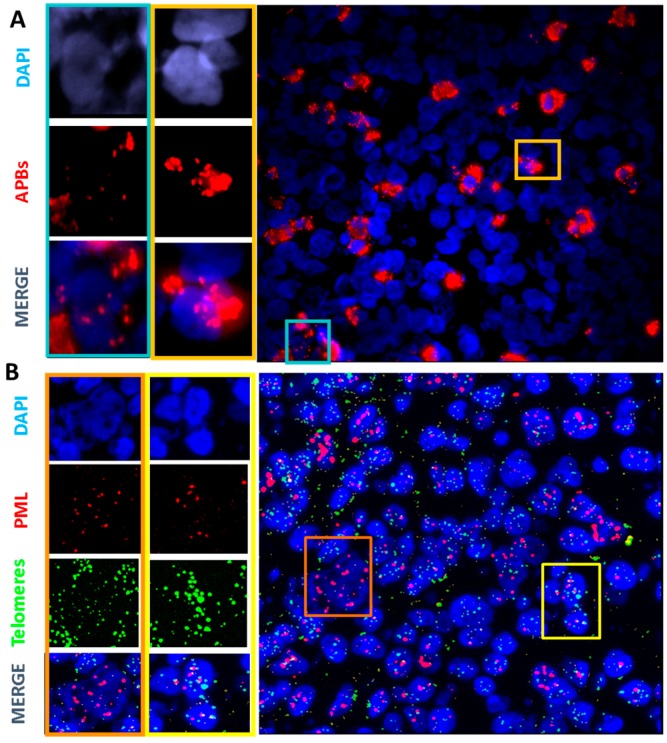
In situ analysis of the presence of APBs in sections of HL lymph nodes. (**A**) Proximity ligation assay (PLA) was used to analyze PML/TRF2 interactions. The yellow frame indicates small cells with multiple and strong signals and blue frame large cells with few signals (40× magnification). (**B**) IF-FISH staining of PML (red) and PNA telomeres (green) show co-localization of PML with telomere sequences in small cells (yellow frame) and high expression of PML in HRS cell with few co-localization signals (orange frame) (40× magnification).

Next, we examined the TMMs of HRS cells from lymph nodes that expressed low or no hTERT. For this analysis, we assessed co-localization of PML and TRF2, forming APBs, as a marker of ALT [29], the unique ALT assay applicable to HL lymph nodes. HL lymph nodes showed high variability in the proportion of cells with high levels of APBs. Using the PLA assay, only one of 24 lymphadenitis lymph nodes displayed co-localization of TRF2 and PML signals in the control group. We observed three levels of APB expression in HL lymph nodes: (a) No expression or low (<5%), (b) intermediate (between 5% to 15%), or (c) high (more than 15%) (Figure 6A). High APB signals were mostly observed in small cells. HRS cells were characterized by high PML expression. These data were also validated using IF-FISH to quantify the co-localization of PML bodies and telomere sequences (Figure 6B).

**Figure 7:**
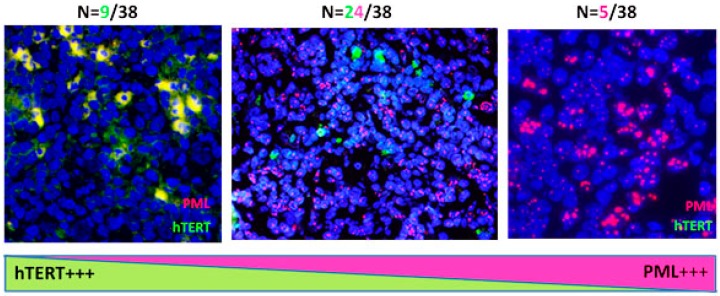
Analysis of the transition between telomerase and ALT in HL lymph nodes from 38 patients. The lymph nodes of nine patients exhibited high levels of telomerase, 24 were characterized by the presence of both telomerase expression and a high level of PML bodies, and five showed a high level of PML bodies with low or undetectable hTERT expression (40× magnification).

Similarly to HL cell lines, HL lymph nodes show coexistence of telomerase and ALT to different degrees. Figure 7 summarizes the results obtained in HL lymph nodes.

### 2.4. TMMs, and Clinical Outcome of HL EBV+ versus EBV− patients

All HL patients (38 patients) entered in this study were stage I (81.6%) or II (18.4%), with a mean age of 36.7 years (84.7% < 45 years). The follow-up of HL patients and controls exceeded 10 years. Four patients (10.5%) died after relapse or refractory or secondary cancer, and four patients (10.5%) developed a late complication (secondary cancer and cardiovascular disease) and are alive with disease; one patient was lost to follow-up. No patients in the control group have died (Table 1).

The EBV status in lymph nodes, assessed by immunostaining of latent membrane protein 1 (LMP1), revealed the presence of the EBV genome in HRS cells in 13 of 38 lymph nodes (34.2%). These data were previously published [30].

Three prognostic groups with low, intermediate, or high telomerase expression and an ALT profile (APBs) were defined with cutoffs of less than 5%, 5% to 15%, and more than 15%, using automated software (Figure S4).

**Figure 8:**
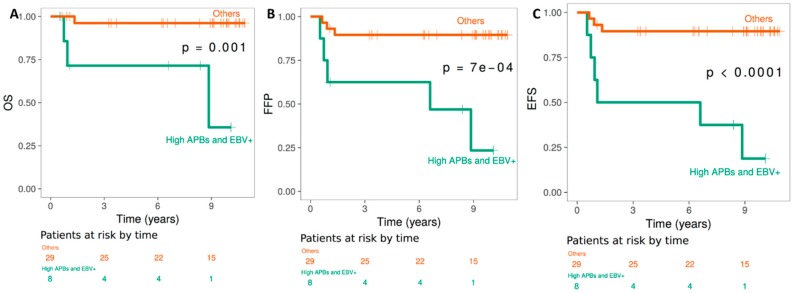
Analysis of the relationship between high expression of APBs in EBV+ HL patients and clinical outcome: (**A**) overall survival, (**B**) freedom from progression, and (**C**) event-free survival.

The association between various markers and survival, as well as freedom from progression and event-free survival was estimated by the Kaplan-Meier method and the log-rank test [31]. There was a significant correlation between ALT profiles and poor event-free survival (EFS) (*p* < 10^−4^), overall survival (OS) (*p* < 10^−3^), and event-free survival (*p* < 10^−3^) (Figure 8) in EBV positive patients. Of note, neither the high expression of PML nor the high expression of hTERT correlated significantly with the clinical outcome of HL patients.

## 3. Discussion

The correlation between telomere dysfunction in the malignant cells [6,32] and clinical outcome of HL patients [5,33,34] has been addressed previously, but with conflicting conclusions concerning TMMs [16,18]. Given the technical difficulties in detecting TA [35], as well as the rarity and heterogeneity of malignant cells in HL lymph nodes, it is perhaps not surprising that the few published telomerase studies have yielded contradictory results [27]. Therefore, we readdressed the issue of TMMs in HL by studying seven HL cell lines and 38 diagnostic lymph-node specimens obtained from HL patients.

First, we assessed TA in HL cell lines using the TRAP assay, followed by PCR. In parallel, we measured hTERT protein levels by immunofluorescence and found a high correlation between TA and the intensity of hTERT protein fluorescence. Thus, immunofluorescence for the detection of hTERT protein offers the possibility to investigate hTERT expression as a surrogate for measuring TA in neoplastic and non-neoplastic cells in HL lymph nodes.

ALT has never been investigated in HL lymph nodes or HL cell lines. The most reliable methods for detecting ALT in tumor samples or cell lines are assays that assess the formation of APBs [10,29], the presence of C-circles [11], the heterogeneity of telomere lengths [36], or the presence of T-SCE. Here, we used the formation of APBs and T-SCE to assess ALT in HL cells [37,38].

In this study, we demonstrate not only the presence of varying levels of TA in all HL cells, but also the co-existence of both TA and ALT in HL cells and the transition between TMMs during progression from small cells to RS cells. Four subpopulations of cells were detected: cells negative for telomerase or ALT, cells with only telomerase expression, cells with only an ALT profile, and cells with both telomerase and ALT profiles. Small cells with a non-neoplastic morphology and with or without CD30 expression, present in variable proportions in HL lymph nodes and HL cell lines, exhibited high telomerase expression than RS cells. We confirmed these data by fluorescence-activated cell sorting after CD30/CD15 staining of HL cell lines. Large numbers of CD30−/CD15− cells exhibited high telomerase expression compared to CD30+/CD15+ cells. Mounting evidence suggests that telomerase is essential for normal stem cell function, and that telomerase expression decreases progressively in their more differentiated progeny [39,40], suggesting that telomerase is the primary factor that regulates normal stem cell function [41,42,43]. The presence of telomerase expression in CD30− cells in HL lymph nodes and HL cell lines suggests that they could represent the “cancer stem” cell of HL [41].

We demonstrated, for the first time, that these small CD30−/CD15− cells with high levels of telomerase subsequently acquired an ALT profile during progression towards the HRS phenotype. It is possible that these cells may activate both pathways for telomere maintenance due to their hypermutability [44], the complexity of DNA damage, and the acquisition of chromosomal instability [44]. Nevertheless, HRS cells, characterized by telomere shortening [45,46] and complex chromosomal rearrangements [47], presented a very low level or absence of telomerase expression, a low level of ABPs compared to small cells, and high level of PML body expression. Thus, three hypotheses are plausible: (1) HRS cells, with their genomic complexity, have reached a state of stability and there is no need for high telomerase or ALT activity for their maintenance. The high expression of PML bodies could contribute to the survival of these cells that exhibit a very low proliferation index [48,49]. (2) Manual scoring of ABPs (PML/PNA telomeres) in HRS cells accurately assesses telomere shortening and telomere deletion in these cells, and the use of a sensitive approach to detect ABPs (PLA assay) may be countered by the possible lack of TRF 2 expression [28,50,51]. (3) Other ALT profiles exist in HRS.

Intriguingly, the presence of cells which have neither TA nor evidence of an ALT profile was found mainly in CD30−/CD15− cells. The presence of malignant cells without any TMMs has been reported previously [15,52]. The molecular basis for this is still unclear. It is possible that another TMM mechanism exists or that these tumor cells do not possess a TMM. This is an important question in the context of TMM as a therapeutic target.

A correlation between TMMs and the clinical outcome of cancer patients has been previously suggested. TA and ALT showed a varied prognosis [15,52,53] reflecting the subtypes of these diseases and the complexity of ALT molecular mechanisms. Here, our data suggest a correlation between clinical outcome and the TMMs in HL patients from a homogeneous cohort of HL patients (81% stage I, more than 94% SN, and young). Lower OS, FFP, and EFS was observed for the ALT profile (ABPs) in EBV+ cases. Moreover, our data suggest that increasing levels of APBs in EBV+ patients are related to poor clinical outcome in our cohort of HL patients. Low numbers of cells with APBs in EBV− patients with early stage defined a large subgroup of patients for whom the rate of long-term disease-specific survival was 100%, following available treatments. No correlation was found between high hTERT expression and PML body expression and clinical outcomes of HL patients. Probably, the small number of patients in this cohort does not allow detection of such correlations. High levels of PML body expression significantly correlated with overall survival in malignant fibrous histiocytoma [54]. However, EBV status appeared to be a prognostic factor and thus confirms several reports on the role of EBV in the clinical outcome of HL patients [55].

Our findings demonstrate the transition and coexistence of both TMMs in the evolution of malignant cells in HL genesis. The ability to explore these processes for the development of novel therapies has so far been limited, due to the lack of in vitro cultivation of primary cells derived from HL tumors. However, the establishment of an HL animal model may provide a major opportunity to shed light on these mechanisms and design novel therapies. Thus, we recently demonstrated, after in vivo transplantation of L428 cells at different stages of antigen acquisition, that the pattern of TMMs evolves, with high telomerase expression in the small CD30−/CD15− cells, and progressive expression of an ALT profile until acquisition of the HRS cell phenotype, with predominant PML bodies, low telomerase expression [56], and the presence of tumor cells without TMM staining. Cytogenetics and molecular characterization of these different cells could advance our insight into not only the mechanisms of the transition and coexistence of telomerase and ALT, but also of the genesis and development of novel therapies in HL. Results from recent clinical trials of telomerase-targeted therapies also underscore the clinical importance of telomere maintenance. Thus, the mosaicism of TMMs within human tumors may affect the choice of therapeutic approaches [57].

Our findings need to be confirmed in large prospective longitudinal clinical studies to better understand the TMMs in HL and establish the relationship between these markers and clinical outcome.

Much progress has been made in understanding telomere structure and function in normal and neoplastic cells [58]. It is critically important to develop reliable methods for telomerase and ALT detection, as they are likely to become important targets in the treatment of cancer patients. A striking correlation has been observed between ALT activity in various human cancers and loss of the ATP-dependent helicase ATRX or its binding partner, the H3.3-specific histone chaperone DAXX, both of which are constituents of PML bodies. A recent study has found that cancer cells with an ALT profile are hypersensitive to ATR inhibitors [59]. Hence, it will be informative to test the in vitro activity of ATR inhibitors in HL cell lines [60] and in vivo using our HL animal model [55].

## 4. Materials and Methods

### 4.1. Patient Samples

Pretreatment biopsies from 38 patients with a histological diagnosis of nodular sclerosis, mixed cellularity, or classical lymphocyte-rich HL were obtained from the Department of Anatomo-Pathology of the University Hospital Centre of Lille. The study was approved by the Institut de Biologie of Lille (CNRS) and University Hospital Centre of Lille Institutional Review Boards, and an informed consent was obtained in writing from each donor (Table 1) [30].

A group of control samples was included in this study consisting of lymph nodes derived from 24 patients (mean age 37.2 years) with follicular hyperplasia or chronic lymphadenitis. Seven HL-derived cell lines (L540, L428, KMH2, HDLM-2, L1236, L591, and SUP-HD1), obtained from the German Collection of Microorganisms and Cells were also included. Cultures and staining results were obtained from three independent cultures of each cell line.

### 4.2. Telomere Repeat Amplification Protocol (TRAP) Assay

The PCR-based telomere repeat amplification protocol (TRAP) assay of telomerase enzyme activity using the TeloTAGGG™ Telomerase PCR ELISA PLUS (Roche-12013789001) was carried out as described previously [8,61] and according to the manufacturer’s instructions. Briefly, cellular extracts were prepared by homogenizing cells in either CHAPS extraction buffer or Buffer C (20 mmol/L HEPES (pH 7.9), 420 mmol/L KCl, 5 mmol/L MgCl_2_, 25% glycerol, 0.1 mmol/L EDTA, 0.2% NP40). Total protein concentrations were determined using a detergent compatible with the protein assay (Bio-Rad, Marnes La Coquette, France). Equivalent amounts of extract corresponding to 1 μg total protein were used for each reaction. Three independent TRAP assays were performed for each extract.

### 4.3. Immunofluorescence Detection of ALT-Associated PML Nuclear Bodies and Shelterin Protein Complexes

Five-μm frozen tissue sections or cultured cells applied onto polylysine coated slides by Cytospin^®^ were fixed with 4% formaldehyde (PFA, Sigma-Aldrich, Saint Quentin Fallavier, France) for 10 min, and treated with 0.25% Triton X-100 solution (Sigma-Aldrich) for 10 min. After blocking with 5% bovine serum albumin (BlockingReagent^®^ Sigma), the cells were then stained with a primary antibody overnight at 4 °C and counterstained with a secondary antibody. Cell nuclei were stained with 4′,6-diamidino-2-phenylindole (DAPI, Sigma-Aldrich). Staining was carried out in the absence of primary antibody as a negative control.

The primary antibodies used were as follows: PML (InterBiotech Interchim, Montliçon, France), hTERT (Sigma Aldrich), TRF2 (Santa Cruz Biotechnology, Santa Cruz, CA, USA) and CD30 (DAKO, les Ulis, France). The secondary antibodies used were as follows: anti mouse Cyanine 3 (1/300 in PBS-2% FCS, Sigma-Aldrich) and anti-rabbit FITC (1/100 in PBS-2% FCS, Sigma-Aldrich).

Proximity Ligation Assay (PLA) tests were performed according to the manufacture’s protocol (Duolink, Olink Bioscience, Uppsala, Sweden) [36]. PLA was used for analyzing the TRF2/PML interaction.

Images of immunofluorescent staining were captured with a charge-coupled device camera (Zeiss, Thornwood, New York, NY, USA) coupled to a Zeiss Axioplan microscope using MetaSystems^®^ software (Altlussheim, Germany), which allows automatic image scanning. MetaCyte (Metasystems^®^) was used to quantify the fluorescence of different signals.

### 4.4. Western Blotting Analysis of Lysates

PML expression was tested by Western blotting on lysates from seven HL cell lines using anti-PML (H238, sc-5621, Santa Cruz Biotechnology). Cells were sonicated in 500 μL of a buffer containing 8 mol/urea, 150 mmol/L b-mercaptoethanol, 50 mmol/L Tris-HCL(pH7.2) and centrifuged for 30 min at 4 °C to remove cellular debris. Samples were submitted to electrophoresis on 12% SDS-polyacrylamide gels, blotted onto nitrocellulose membranes and developed using the ECL system (Amersham, Uppsala, Sweden). To verify that equivalent amounts of each sample were loaded, the filters were additionally probed with GAPDH antibody (Santa Cruz Biotechnology). Densitometry was performed to evaluate the intensity of PML and GAPDH bands.

### 4.5. Fluorescence-Activated Cell Sorting and Flow Cytometry

Cells were gated to exclude apoptotic or necrotic cells and sorted into CD30−/CD15−, CD30+ and CD15+/CD30+ fractions by gating on the lowest and highest 5% PE-expressing cells, respectively. Following sorting, the CD30−/CD15− cell fractions were analyzed using a FACScan flow cytometer (Becton Dickinson, Franklin Lakes, NJ, USA) and found to be more than 98% pure. For phenotypic analyses of cell lines or sorted cells, cells were prepared as described, and then stained with mouse anti-human CD30—phycoerthrin (PE) and CD15—fluorescein isothyocyanate (FITC), (all antibodies from BD PharMingen, San Diego, CA). Cells were subsequently analyzed using a FACS LSRII (Becton Dickinson).

### 4.6. Cytogenetics Analysis

#### 4.6.1. FISH Technique

FISH was performed using a combination of standard procedures from the recommended protocols for chromosome analysis using specific probes for the PML-RARα and hTERT genes. The copy number and localization of each gene was assessed and 100 metaphases were scored per cell line. Q-FISH was performed using a Cy-3-labelled PNA probe specific for (TTAGGG) telomere sequences (Eurogenetec, Liege, Belgique) as described previously [33].

#### 4.6.2. CO-FISH

Cells were incubated for 24 to 48 h in fresh medium containing BrdU (10 µg/mL). One hour before harvesting, Colcemid was added to the media to induce the accumulation of mitotic cells. Cells were harvested and resuspended in 0.075 M KCl (pre-warmed to 37 °C) and ice-cold methanol-acetic acid (3:1 ratio) was added to the cell suspension. The cell suspension was centrifuged (5 min at 1000 rpm) and washed twice in methanol-acetic acid. Cells were dropped onto slides and allowed to dry overnight. Slides were rehydrated in 1× PBS for 5 min at room temperature, incubated with 0.5 µg/mL RNase A (in PBS, DNase free) for 10 min at 37 °C, and stained with 0.5 µg/mL Hoechst 33258 in 2× SSC for 15 min at room temperature. Subsequently, slides were placed in a shallow plastic tray, covered with 2× SSC, and exposed to 365 nm ultraviolet light at room temperature for 45 min. The BrdU-substituted DNA strands were digested with at least 10 U/µL of Exonuclease III at room temperature for 30 min. Slides were washed in 1× in PBS, dehydrated in an ethanol series of 70%, 95%, and 100% and air dried. FISH was performed as described above, except that slides were not subjected to DNA denaturation.

#### 4.6.3. IF-FISH

In order to co-localize the PML bodies and telomere sequences, IF-FISH was performed. Following fixation (3% PFA, 2% sucrose), Five-μm frozen tissue sections or cultured cells applied onto polylysine coated slides by Cytospin^®^ were fixed with 4% formaldehyde (PFA, Sigma-Aldrich) for 10 min, and treated with 0.25% Triton X-100 solution (Sigma-Aldrich) for 10 min. cells were immunostained as already described. Cells were successively fixed (PFA 4%, 2 min), washed in PBS, dehydrated (50/70/100 ethanol), and the telomeres hybridized with the PNA probe (CCCTAA) 3-FITC.

#### 4.6.4. Statistical Analysis

The follow-up time was defined as the time from biopsies to either last follow-up or a given event. Event-free survival (EFS) was measured from the date of biopsies to either disease progression or discontinuation of treatment for any reason or censoring date. Overall survival (OS) was measured from the date of diagnosis to the date of death for any reason. Three groups were considered for survival analysis associated with TRF2 and PML co-localization, PML or hTERT expression: The tertile of patients with the highest (>15%), intermediate (between 5% to 15%), and lowest expression (<5%). These cutoff points were chosen in accordance with previous reports. Survival was estimated by the Kaplan-Meier method and compared using a log-rank test [31]. Two-sided *p*-values < 5% were considered to be statistically significant and *p* < 10% to be borderline significant.

## 5. Conclusions

Using multiple approaches, we find that HL cells maintain their telomeres by the concomitant use of both telomerase and ALT during their evolution, reflecting tumor heterogeneity in HL. Furthermore, the mosaicism of TMMs in HL may have important implications in the treatment of this disease.

## Figures and Tables

**Table 1:** HL Patient’s characteristics.

Characteristics	No. of Patients (*n* = 38)	%
**Male**	19	50
**Age**	36.7 y	
<45	32	84.2
>45	6	15.8
**Stage**		
Stage I	31	81.6
Stage II	7	18.4
**Treatment**		
Chemotherapy only	0	0
Combined modality	38	100
**Histological sub-type**		
Nodular Sclerosis	36	94.7
Mixed cellularity	1	2.6
Classic. lymphocyte rich	1	2.6
**EBV+(LPM1)**	13	34.2
**Follow-up**		
ACR	30	78.9
AWD	4	10.5
DOD	4	10.5

ACR: Alive in complete remission; AWD: Alive with disease; DOD: Dead of disease.

## Supplementary Materials

The following are available online at http://www.mdpi.com/2072-6694/10/6/169/s1, Figure S1: Metaphases depicting copy number of hTERT (red) in L428 (higher TA) and L1236 (lower TA) cells. hTERT (5p15) copy number and control EGR1/CDC25C (5q31) are presented. Figure S2: Metaphases depicting copy number of PML (red) in L428 and L1236 cells. PML (15q22) copy number and the number of chromosome 15 are presented. Figure S3: Positive control of hTERT and PML expression in fibroblast derived from cancer patient showing the cytoplasmic signal of hTERT and nuclear signal from PML bodies. Figure S4: Three prognostic groups of ALT profile with high, intermediate, low or negative APBs detected using PLA assay, were assessed.
