# Supplementary material for: The Transition between Telomerase and ALT Mechanisms in Hodgkin Lymphoma and Its Predictive Value in Clinical Outcomes
Source: Cancers (Basel). 2018 May 30;10(6):169. doi: 10.3390/cancers10060169 (PMC6025489; doi:10.3390/cancers10060169)
Supplement: Supplementary file 1 [file cancers-10-00169-s001.pdf]

Supplementary Materials: The Transition between Telomerase and ALT Mechanisms in Hodgkin Lymphoma and Its Predictive Value in Clinical Outcomes

Radhia M'kacher, Corina Cuceu, Mustafa Al Jawhari, Luc Morat, Monika Frenzel, Grace Shim, Aude Lenain, William M. Hempel, Steffen Junker, Theodore Girinsky, Bruno Colicchio, Alain Deterlen, Leonhard Heidingsfelder, Claire Borie, Noufissa Oudrhiri, Annelise Bennaceur-Griscelli, Olivier Moralès, Sarah Renaud, Zoé Van de Wyngaert, Eric Jeandidier, Nadira Delhem and Patrice Carde

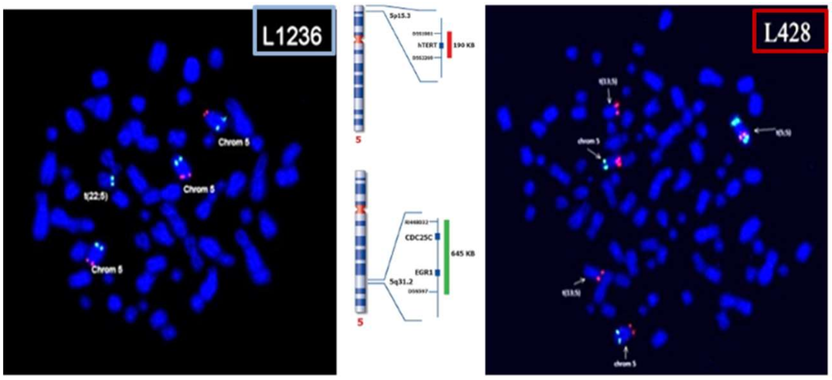

Normal Signal pattern	2/ 2
Cell lines	hTERT/ control 5p15/5q31.1
L428	5/4
SUP-HD1	2/2
L540	4/3
KM-H2	2/2
L591	2/2
HDML-2	3/2
L1236	3/4

Figure S1. Metaphases depicting copy number of hTERT (red) in L428 (higher TA) and L1236 (lower TA) cells (63× magnification). hTERT (5p15) copy number and control EGR1/CDC25C (5q31) are presented.

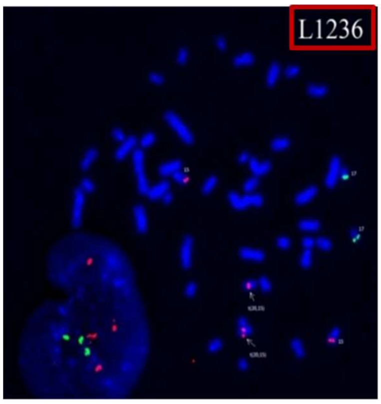

Normal Signal	2	Nb chr 15
Cell line	PML gene (15q22)	
L428	4	4
L540	3	3
L591	2	2
SUP-HD1	2	2
HDML-2	2	1
KM-H2	4	3
L1236	4	3

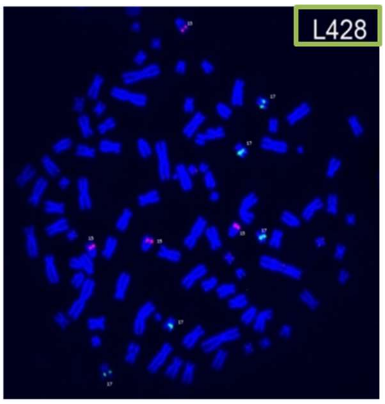

Figure S2. Metaphases depicting copy number of PML (red) in L428 and L1236 cells (63× magnification). PML (15q22) copy number and the number of chromosome 15 are presented.

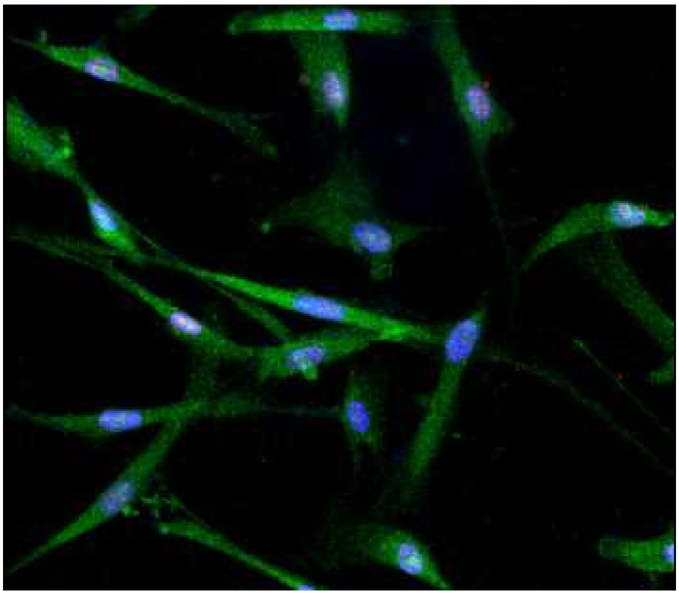

Figure S3. Positive control of hTERT and PML expression in fibroblast derived from cancer patient showing the cytoplasmic signal of hTERT and nuclear signal from PML bodies (40× magnification).

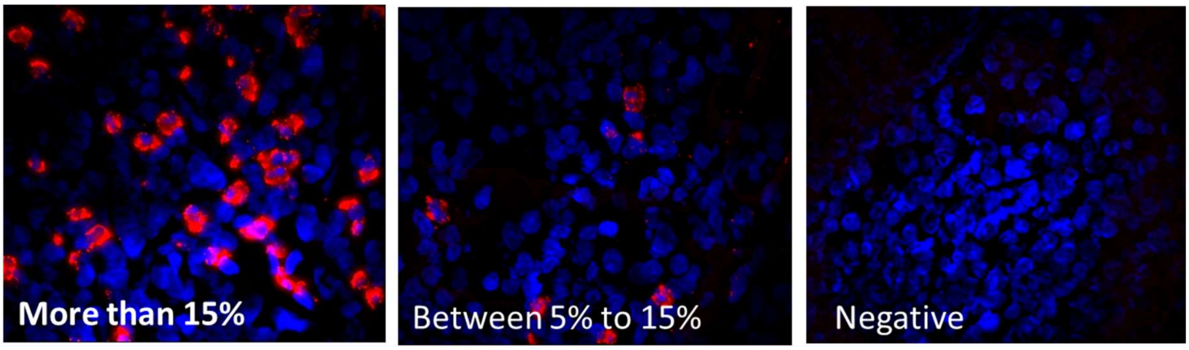

Figure S4. Three prognostic groups of ALT profile with high, intermediate, low or negative APBs detected using PLA assay, were assessed (40× magnification).

© 2018 by the authors. Licensee MDPI, Basel, Switzerland. This article is an open access article distributed under the terms and conditions of the Creative Commons Attribution (CC BY) license (http://creativecommons.org/licenses/by/4.0/).
